# Supplementary material for: Deep learning-based model for detecting 2019 novel coronavirus pneumonia on high-resolution computed tomography
Source: Sci Rep. 2020 Nov 5;10:19196. doi: 10.1038/s41598-020-76282-0 (PMC7645624; doi:10.1038/s41598-020-76282-0)
Supplement: Supplementary file 1 — Supplementary Legends. [file 41598_2020_76282_MOESM1_ESM.docx]

SUPPLEMENTARY FIGURES

Supplementary Figure 1. The training curves of UNet++ for extracting valid areas in Computed tomography images.

Supplementary Figure 2. The training curves of UNet++ for detecting suspicious lesions in Computed tomography images.
