# Supplementary figures and images for: Deep learning-based model for detecting 2019 novel coronavirus pneumonia on high-resolution computed tomography
Source: Sci Rep. 2020 Nov 5;10:19196. doi: 10.1038/s41598-020-76282-0 (PMC7645624; doi:10.1038/s41598-020-76282-0)

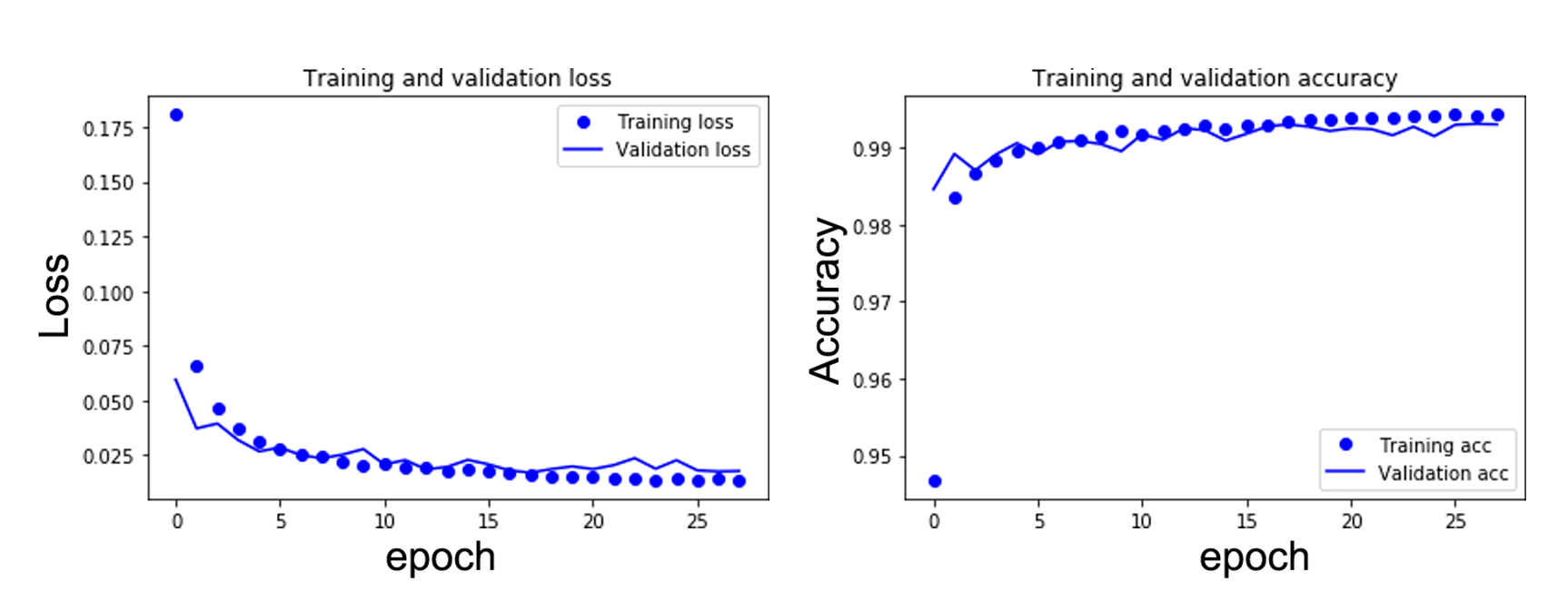

Supplement: Supplementary file 2 — Supplementary Information 2. [file 41598_2020_76282_MOESM2_ESM.tif]

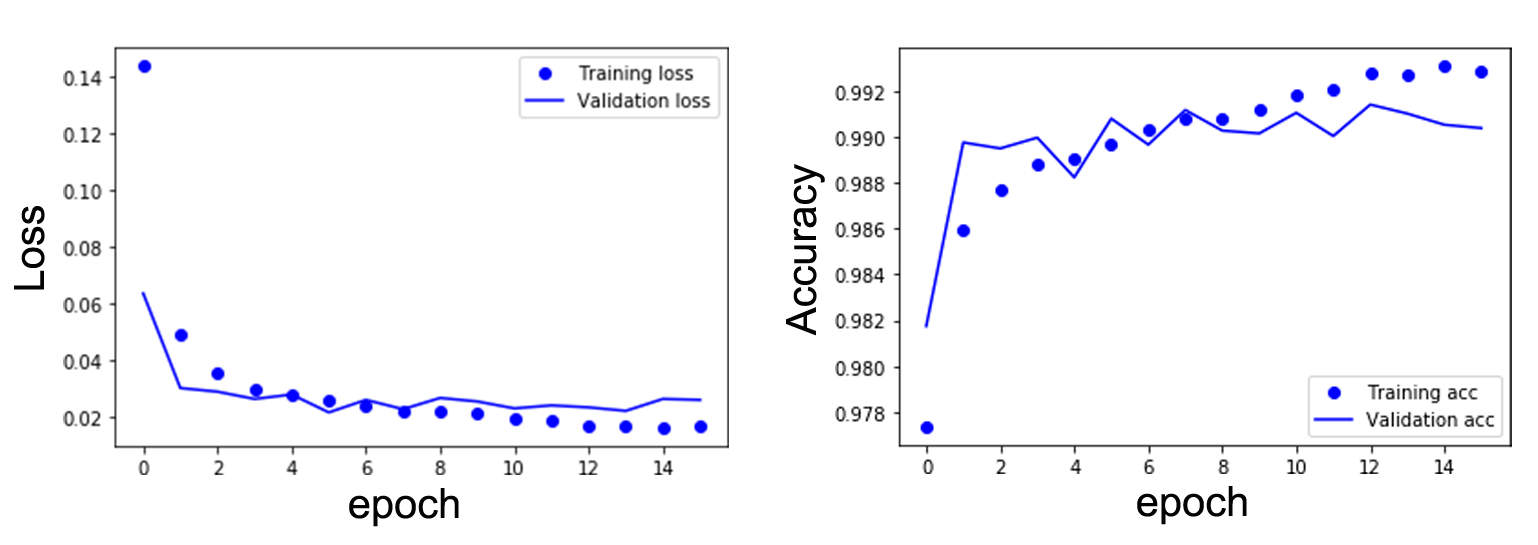

Supplement: Supplementary file 3 — Supplementary Information 3. [file 41598_2020_76282_MOESM3_ESM.tif]
